# Ceftobiprole Perspective: Current and Potential Future Indications

**DOI:** 10.3390/antibiotics10020170

**Published:** 2021-02-08

**Authors:** Tommaso Lupia, Carlo Pallotto, Silvia Corcione, Lucio Boglione, Francesco Giuseppe De Rosa

**Affiliations:** 1 Infectious Diseases Unit, Cardinal Massaia Hospital, 14100 Asti, Italy; francescogiuseppe.derosa@unito.it; 2 Infectious Diseases Unit 1, Santa Maria Annunziata Hospital, Central District, Tuscany Health Care, Bagno a Ripoli, 500012 Florence, Italy; pallottoc@gmail.com; 3 Department of Medical Sciences, Infectious Diseases, University of Turin, 10124 Turin, Italy; corcione.silvia@gmail.com; 4 Infectious Diseases, Tufts University School of Medicine, Boston, MA 02109, USA; 5 Department of Translational Medicine, University of Eastern Piedmont, 28100 Novara, Italy; lucio.boglione@uniupo.it

**Keywords:** ceftobiprole, cephalosporin, pneumonia, bloodstream infections, stewardship

## Abstract

Ceftobiprole combines an excellent spectrum for community-acquired pneumonia (CAP) and hospital-acquired pneumonia (HAP) pathogens, with a low/medium MDR risk, and the β-lactams’ safety in frail patients admitted to the hospital in internal medicine wards which may be at high risk of adverse events by anti-MRSA coverage as oxazolidinones or glycopeptides. We aimed to report the available evidence regarding ceftobiprole use in pneumonia and invasive bacterial infections, shedding light on ceftobiprole stewardship. The clinical application and real-life experiences of using ceftobiprole for bloodstream infections, including infective endocarditis, are limited but nevertheless promising. In addition, extended-spectrum ceftobiprole activity, including *Enterococcus faecalis*, *Enterobacteriaceae*, and *Pseudomonas aeruginosa*, has theoretical advantages for use as empirical therapy in bacteremia potentially caused by a broad spectrum of microorganisms, such as catheter-related bacteremia. In the future, the desirable approach to sepsis and severe infections will be administered to patients according to their clinical situation, the intrinsic host characteristics, the susceptibility profile, and local epidemiology, while the “universal antibiotic strategy” will no longer be adequate.

## 1. Introduction

Ceftobiprole medocaril, a fifth-generation extended-spectrum cephalosporin, is endorsed in Europe at a standard dosage of 500 mg intravenously every 8 h for managing adult community-acquired pneumonia (CAP) [1], non-ventilator-associated hospital-acquired pneumonia (HAP) [2], and skin and soft-tissue infections (SSTIs) [3,4], including diabetic foot infections. Ceftobiprole was the first β-lactam antibiotic to show in vitro activity against methicillin-resistant *Staphylococcus aureus* (MRSA), vancomycin-resistant *S. aureus* (VRSA), and penicillin-resistant *Streptococcus pneumoniae* (PRSP) [5]. It exerts its antibacterial activity by inhibiting the transpeptidase moiety of penicillin-binding proteins (PBPs) [6]. More precisely, ceftobiprole’s in vitro activity demonstrates potent binding against PBPs of Gram-positive bacteria (GPB), including those with decreased β-lactam sensitivity, such as PBP2x and PBP2bin PRSP and, PBPa, which confers methicillin resistance to *S. aureus* strains [5,6]. In vitro bactericidal activity against MRSA strains showed kinetics either similar or superior to those of vancomycin and linezolid [7]. Furthermore, recent findings regarding isolates from ceftobiprole phase III SSTIs and pneumonia clinical trials showed promising activity on MRSA isolates, including Panton–Valentine-leukocidin-positive strains, regardless [8] or with a slight variation [9] according to SCC*mec* or clone type.

Ceftobiprole retains activity against a wide spectrum of Gram-negative bacteria (GNB) and is stable against a wide variety of β-lactamases, being similar to ceftriaxone, cefepime, and ceftazidime [10]; the class A β-lactamases PC1 staphylococcal penicillinase, TEM, some SHV types, and the K1 β-lactamase of *Klebsiella oxytoca* have no lytic enzymatic action against ceftobiprole. The drug is degraded by both extended-spectrum β-lactamases (ESBLs) and serine-carbapenemases. Class B, several class C chromosomal AmpC-type β-lactamases, and some class D β-lactamases have lytic action on ceftobiprole’s structure [5,6].

Since several reports are available regarding bloodstream infections, including infective endocarditis, we aimed to report the available evidence regarding ceftobiprole use for the approved indications and potential further indications, shedding light on ceftobiprole stewardship.

### 1.1. Ceftobiprole and Pneumonia

Ceftobiprole’s efficacy and safety have been demonstrated through two phase III trials [1,2]. Nicholson et al. designed a non-inferiority, double-blinded, multicenter, randomized trial where ceftobiprole was compared against ceftriaxone with optional linezolid (if a high risk of MRSA or ceftriaxone-resistant *S. pneumoniae* were present) at regular dosages under extreme CAP [1]. The non-inferiority of ceftobiprole was confirmed in the previous study and compared to the linezolid combination therapies. The second (double-blinded, multicenter, and randomized) study compared ceftobiprole medocaril and ceftazidime combined with linezolid within HAP and ventilator associated pneumonia (VAP). Awad et al.’s study showed the non-inferiority of ceftobiprole against ceftazidime plus linezolid, except in patients with VAP [2]. The failure of ceftobiprole to achieve non-inferiority in VAP subgroups might be related to insufficient sample size and higher heterogeneity in VAP patients according to demographic, clinical, and microbiological characteristics.

Furthermore, another significant statistical difference was seen in the subgroup of those with microbiological evidence of MRSA infection, with 94.7% in the ceftobiprole group vs. 52.6% in the ceftazidime plus linezolid group, reflecting a discrepancy of 42.1% (95% CI, 17.5–66.7%). Regarding the secondary efficacy criterion, the rates of microbiological eradication were comparable in the ceftobiprole and ceftazidime/linezolid groups at the management visit’s end for patients who had HAP [2]. Interestingly, the clinical results were in favor of ceftobiprole in patients having HAP and needing mechanical ventilation (MV) for less than 48 h [2,10,11].

In severe CAP leading to influenza-associated complications, ceftobiprole can be an excellent option to ensure that realistic coverage of community-acquired MRSA can be assured, especially in patients with diabetes, obesity, or chronic obstructive pulmonary disease (COPD), as well as patients over 65 years of age with pulmonary anomalies or with underlying malignancies [5,10,11].

In a post hoc review of both phase III trials [1,2], Scheeren et al. [12] evaluated the consequences for a limited group of high-risk patients with community-acquired or nosocomial pneumonia, and found ceftobiprole to be compelling relative to other drugs regarding premature improvement in the treatment of high-risk patients, patients with severe HAP, and patients with up to ten underlying comorbidities.

Nosocomial pneumonia recommendations [13,14] include rapid empirical antimicrobial treatment regimen using a combination of local antibiotic resistance, risk factors and severity of disease.

If the risk of multi-drug-resistant (MDR) bacterial etiology is low to medium, initial monotherapy can be used wherever possible [15,16]. Ceftobiprole blends an excellent spectrum of low- to medium-MDR-risk HAP pathogens with β-lactam protection in vulnerable, hospitalized patients at high risk of adverse reactions triggered by anti-MRSA coverage, such as oxazolidinones or glycopeptides. In addition to the above characteristics, ceftobiprole is at low risk for the selection of resistant GPB or GNB mutants, and does not have any significant effect on the stable human intestinal flora.

Besides the above-mentioned qualities, ceftobiprole has minimal risk for the selection of resistant mutants in GPB or GNB, and no significant effects on stable human intestinal flora [17,18].

**Figure 1:**
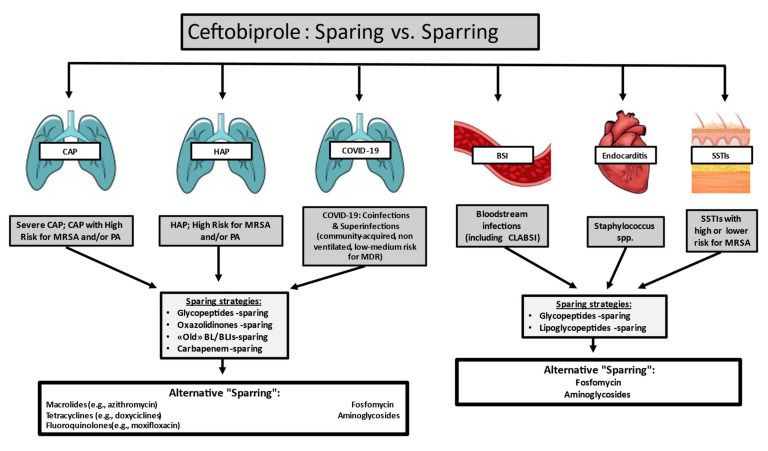
Ceftobiprole stewardship perspective: sparing vs. sparring. Abbreviations: CAP—community-acquired pneumonia; MRSA—methicillin-resistant *Staphylococcus aureus*; PA—*Pseudomonas aeruginosa*; HAP—hospital-acquired pneumonia; CLABSI—central-line associated bloodstream infections; BL/BLI—β-lactams/β-lactam inhibitor; SSTIs—skin and soft tissue infections.

As of 18 January 2021, the SARS-CoV-2 pandemic has caused 90.5 million cases and 2.0 million deaths worldwide [19,20]. Data are increasing regarding bacterial co-infections in the severe disease caused by SARS-CoV-2, but the subject is not yet fully understood [21,22]. GPB are the most common pathogens that cause community-acquired pneumonia co-infections in COVID-19 subjects, although there is an increasing ratio of infections with GNB among hospital-acquired lung superinfections [21,22,23,24,25,26,27,28]. Ceftobiprole may fit within a monotherapy stewardship intervention in patients with CAP or HAP at risk for MRSA or *Pseudomonas aeruginosa* [29], with or without subsequent de-escalation (Figure 1).

### 1.2. Ceftobiprole and Skin and Soft Tissue Infections

Regarding the use of ceftobiprole within SSTIs, three randomized clinical trials [3,4,30] have confirmed the drug’s usefulness, although a permit to use was subsequently not issued [31]. In subjects who were suspected of or confirmed to have complicated Gram-positive SSTIs, Noel et al. published the first phase III, non-inferiority, double-blind, randomized clinical trial (RCT) at 129 sites worldwide [3]. In patients, either ceftobiprole at 500 mg for 12 h, or vancomycin at 1 g for 12 h, was randomized [3]. The therapy lasted 7–14 days. Infections of the diabetic foot, bite-wound, and osteomyelitis were removed [3]. The key endpoint of the visit to the test of cure (TOC) was a therapeutic cure (7–14 days after the treatment) [3]. The margin for non-inferiority was set at 10%. For the Intention-To-Treat (ITT) population 77.8% (308/397) and 77.5% (300/387) respectively of ceftobiprole-treated and vancomycin-treated patients (difference 0.3%, 95% CI, −5.5%, and 6.1%) were treated with the clinical cure [3]. In the population, 93.3% (263/282) and 93.5% (259/277) of the ceftobiprole and vancomycin treated patients were clinically cured, (difference −0.2%, 95% CI, −4.4% to 3.9) respectively [3].

In patients with complex Gram-positive or Gram-negative SSTIs, a second double-blind RCT comparing ceftobiprole to vancomycin plus ceftazidime was performed [31]. During the TOC visit, the critical endpoint was a therapeutic cure (7–14 days after the treatment) [4]. The 10% margin for non-inferiority was set [4]. Noel et al. showed that a clinical cure was reached in the ITT population in 81.9% (448/547) and 80.8% (227/281) of ceftobiprole and vancomycin plus ceftazidime treatment groups respectively [4]. Clinical cures were reached in 90.5% (439/485) and 90.2% (220/244) of CE patients treated with ceftobiprole and vancomycin plus ceftazidime, respectively [31].

Overcash et al. [30] recently published the results for treatment of SSTI with vancomycin + aztreonam (*n* = 344) in a novel phase III, double-blind RCT (TARGET trial). In the ceftobiprole and vancomycin + aztreonam groups respectively, early clinical success rates of 91.3% and 88.1%, and non-inferiority were seen [30]. At the TOC visit, the investigator-assessed clinical performance of both groups was comparable and the ITT (90.1% vs 89.0%), as well as those scientifically estimated (97.9% vs. 95.2%), showed non-inferiority [30]. Both treatment groups had similar microbiological results and safety profiles [30].

### 1.3. Ceftobiprole and Bacteremia

According to existing recommendations and research, the mainstays of antibiotic treatment for Gram-positive invasive infections are glycopeptides (e.g., vancomycin and teicoplanin) and lipoglycopeptides (e.g., daptomycin), and some β-lactam antimicrobials (e.g., cefazoline) [31,32,33,34]. For MRSA, the treatment of bloodstream infections (BSIs) and infective endocarditis (IE) is only approved for vancomycin and daptomycin [35,36]. One of the main drawbacks of β-lactams in *Staphylococcus aureus* bacteremia (SAB) treatments is the absence of MRSA action [31,32,33,34]. Currently, ceftaroline is the only β-lactam antibiotic used for MRSA bacteremia and this is off-label with insufficient clinical evidence to provide optimal dose guidance [37,38].

Vancomycin is not as effective as β-lactams against methicillin-susceptible *S. aureus* (MSSA) [39,40,41]. With its bactericidal activity against both MSSA and MRSA, ceftobiprole has the potential to address this shortcoming [42,43,44]. The clinical application and real-life experiences of using ceftobiprole for BSIs are limited but nevertheless promising [45,46]. Phase III trials for CAP, HAP, and SSTIs included a small proportion of Gram-positive bacteremic patients [1,2,3,4]. By extrapolating clinical data directly from phase III trials [1,2,3,4], Rello et al. [46] developed two interesting pooled analyses—a test of cure and mortality—for ceftobiprole versus comparators (e.g., vancomycin, linezolid, and ceftazidime) in BSIs caused by *Staphylococcus* spp. All BSI episodes had as origin either SSTIs or pneumonia and therefore ceftobiprole was used within approved boundaries [46]. The clinical cure rate of the ceftobiprole group (48.9%, 22/45 patients) in the TOC analysis was comparable to that of the comparators (44.0%, 22/50) for staphylococcal bacteremia, specifically the subgroups of coagulase-negative staphylococci (CoNS) (45.5% vs. 45.5%), MSSA (44.4% vs. 46.7%), and MRSA (55.6% vs. 22.2%) [45,46]. Furthermore, the ceftobiprole group showed better results for the 30-day all-cause mortality than its comparators; mortality in the ceftobiprole group was 8.9% (4/45) versus 16.0% (8/50) in the comparators group [45,46]. It is worth noting that the mortality rate for MRSA bacteremic patients was around 0% in the ceftobiprole group versus 22.2% in the comparator cohorts [45,46].

Recently, Durante-Mangoni et al. [47] reported ceftobiprole use in their case series of BSI in 10 patients (34.5%), including three with infective endocarditis (10.3%) and two with skin and skin structure infection (6.9%). The majority of BSIs were due to MRSA (35%) and coagulase-negative staphylococci (35%, mostly methicillin-resistant CoNS) with high severity at presentation (58.6% and 13.8% sepsis and septic shock, respectively) [47]. Nevertheless, the BSI cohort (*n* = 10) by Durante-Mangoni and colleagues presented four failures (three bacteremia and one IE) [47]. We are waiting hopefully for interesting data from some ongoing studies regarding SAB. Recruitment for the first randomized, double-blind, multicenter study comparing ceftobiprole medocaril to daptomycin in the treatment of SAB (ERADICATE trial, https://clinicaltrials.gov/ct2/show/NCT03138733) began in August 2018 [48]. The ERADICATE trial would establish the efficacy and safety of this novel cephalosporin compared to daptomycin (6 mg/kg with or without aztreonam in the treatment of *Staphylococcus aureus* bacteremia, including IE) [48]. The target completion of this study is in the second half of 2021 [48]. In addition, extended-spectrum ceftobiprole activity, including *Enterococcus faecalis*, *Enterobacteriaceae*, and *Pseudomonas aeruginosa*, has theoretical advantages for use as empirical therapy in bacteremia potentially caused by a broad spectrum of microorganisms, such as catheter-related bacteremia [45,46].

### 1.4. Ceftobiprole and Infective Endocarditis

IE is a severe disease with high morbidity and mortality. In the last few decades, the outcome of IE has not significantly improved [49,50]. *Staphylococcus aureus* and other Gram-positive bacteria are the most frequent aetiologic agents; Gram-negative bacteria account for just 4–6% of cases, and they are generally associated with poor outcomes [51,52].

Since it became available, particular interest has been focused on ceftobiprole beyond the approved indications and, in particular, on IE, probably because of (i) its broad-spectrum activity, including Gram-positive and Gram-negative bacteria, especially both MSSA and MRSA; (ii) its bactericidal activity; and (iii) its high tolerability [5]. Animal models of IE treated with ceftobiprole have provided promising results despite being limited mostly to staphylococcal IE. In rats with experimental endocarditis due to two different strains of MRSA, ceftobiprole was able to sterilize >90% of cardiac vegetations [53]. Furthermore, in a rabbit model of MRSA experimental endocarditis, ceftobiprole was compared to vancomycin, linezolid, and daptomycin, and it was described as superior in reducing the microbial burden in aortic valve vegetations [54]. When administered in a combination regimen, ceftobiprole and vancomycin showed a synergistic effect against MRSA and glycopeptide-intermediate *S. aureus* [55], even at reduced dosages [56].

Unfortunately, clinical experience in this field is still scarce, and most of the available data come from in vitro studies and animal models, besides the previously reported data from Durante-Mangoni et al. [47]. Since 2007, ceftobiprole has been described as bactericidal against both MSSA and MRSA isolates from patients with IE [56]. More recently, Rodriguez-Garcia et al. [57] analyzed the susceptibility to ceftobiprole of 77 strains of Gram-positive bacteria recovered from patients with IE. All 40 staphylococcal isolates (11 MSSA, 7 MRSA, 8 methicillin-susceptible coagulase-negative staphylococci, and 14 methicillin-resistant CoNS) were susceptible to ceftobiprole, and 18/18 streptococci had an MIC of about 0.125 μg/mL. Moreover, ceftobiprole showed good results against *Enterococcus faecalis* (MIC90 0.5 μg/mL) [58].

Clinical data from human cases of IE treated with ceftobiprole are still scarce, limited to a few case reports and a small case series. The first case involved a hematopoietic stem cell transplanted patient who was diagnosed with an infection of the prosthesis of the ascending aorta and aortic valve due to methicillin-resistant *Staphylococcus epidermidis*, treated with long-course ceftobiprole monotherapy with clinical and microbiological success [59]. A second case was described in a case series by Mahmoud et al. [60] in which a 74-year-old man with MRSA native mitral valve endocarditis was treated with a 76-day course of ceftobiprole reported a follow-up negative for both endocarditis and microbiological culture. Recently, Tascini et al. [61] published a multicenter case series of 12 patients with IE treated with ceftobiprole, including a case previously described by Oltolini et al. [62]. All the patients were diagnosed with staphylococcal IE and 8/12 had IE in prosthetic valves. Ceftobiprole was initiated after the failure of the previous antibiotic regimens in 9/12 patients; three patients, in particular, had persistently positive blood cultures, which became rapidly negative after ceftobiprole administration. In just one case, ceftobiprole was administered as monotherapy. The remaining 11 patients that featured all *S. aureus* strains were treated with a combination of ceftobiprole and daptomycin that was previously shown to be synergistic in vitro [63]. The cure rate was 83% (10/12); two patients had poor outcomes as a result of fatal arrhythmias. It is of note that the blood cultures of both patients were negative before the exitus.

Ultimately, ceftobiprole is an intriguing possibility for the treatment of endocarditis, especially in combination with daptomycin, but further evidence is needed both from human studies and in vivo and in vitro studies, which should also consider streptococcal and enterococcal etiologies. New data about ceftobiprole versus daptomycin in *S. aureus* bacteremia will be provided by the ongoing trial NCT03138733 [48], which also includes right-sided IE. Meanwhile, the evidence is growing for bacteremia and endocarditis, special interest may be given to ceftobiprole use in the setting of possible or probable Central-Venous Catheter (CVC)-associated BSI [63,64].

## 2. Discussion

Cephalosporins have evolved greatly in the past decade through the creation of new-generation molecules with wide spectrum activity. Ceftobiprole medocaril has been approved for the treatment of adult CAP and HAP (excluding ventilator-acquired pneumonia) in 12 European countries, as well as Canada and Switzerland.

Initial empiric monotherapy with ceftobiprole in pneumonia can be put to use wherever possible in cases of low to medium risk of the growth of MDR bacteria [15,16,29]. Ceftobiprole blends an excellent spectrum for HAP pathogens with low to medium MDR risk with β-lactam safety in frail hospital inpatients, who may be at significant risk of adverse effects triggered by MRSA and anti-MRSA coverage, including oxazolidinones or glycopeptides [15,16,29]. Moreover, in severe adult CAP and post-influenza bacterial pneumonia or COVID-19 pulmonary superinfections, this novel cephalosporin may be a suitable option, notably in subjects at risk for MRSA or *P. aeruginosa* isolates [15,16,29].

Three randomized clinical trials stated that ceftobiprole was helpful in SSTI patients, although a permit to use was subsequently not issued in all countries [30,31,32,33]. Recent findings from ceftobiprole in the first two phase III RCTs on SSTIs also showed a promising activity on Panton–Valentine-leukocidin-positive strains, regardless of SCCmec or clone type [8,9].

In addition, little is known regarding the penetration of cerebrospinal fluid (CSF) for ceftobiprole. Stucki et al. [65] found that ceftobiprole penetration was approximately 16% in inflamed meninges, compared to approximately 2% in uninflamed meninges in a model for rabbit meningitis. In primary meningitis caused by *S. pneumoniae* (including strains resistant to penicillin and ceftriaxone) and the secondary post-operatory meningitis that requires both Gram-positive and Gram-negative coverage, ceftobiprole may be a beneficial option.

The clinical application and real-life experiences of using ceftobiprole for BSIs are limited but nevertheless promising. However, so far data on ceftobiprole in bacteremia patients should be interpreted with caution, and a multi-level assessment from microbiological, pharmacological, clinical, and economic perspectives should be pursued, with consideration for the future of antimicrobial stewardship in BSIs [45,46,47,48,49].

The in vitro microbiological susceptibilities of ceftobiprole are altogether quite promising, although the literature shows variability in the susceptibility rates among diverse Gram-negative and -positive strains from various countries and cohorts [45,46,47,48,49]. This variability makes it crucial to know their epidemiology, particularly in multi-drug-resistant organisms with limited treatment options.

In addition to these favorable MICs, more pharmacological data are needed to assess the best dosage in critically ill patients to avoid administering subtherapeutic drug levels. These new compounds theoretically permit a sparing approach in various antimicrobial classes, such as glycopeptides, lipoglycopeptides, and aminoglycosides, as well as in endovascular infections and bacteremia, and they favor molecules with a good safety profile (Figure 1). The increasing data on ceftobiprole will enable a shift in the management of MRSA and endovascular bacteremia [45,46]. These molecules can also be used in therapeutic combinations, given that combination therapy is effective in particular circumstances. Two different types of antibiotics with one pathogen activity can mainly be used to facilitate the clearance of pathogens and to make sure the pathogen is susceptible to empiric therapy [66,67,68]. Monotherapy, however, decreases the risks of antibiotic pressure, the incidence of new infections, antimicrobial antagonism, toxicity, and costs [68,69]. Combination antibiotic treatment was recommended in the 2016 Surviving Sepsis Campaign Guidelines for the primary management of septic shock to ensure appropriate empiric antibiotic coverage in a situation where MDR pathogens are at great risk [69,70]. However, several other studies have found little benefit to a combination treatment and some analyses have shown that patients who undergo combination therapy have an increased risk of side effects [70,71]. In the future, patients will be treated in accordance with their clinical conditions, inherent host features, susceptibility profile, and local epidemiology, as the “universal antibiotic strategy” is no longer appropriate in the desirable approach to serious infection.

## 3. Materials and Methods

**Figure 2:**
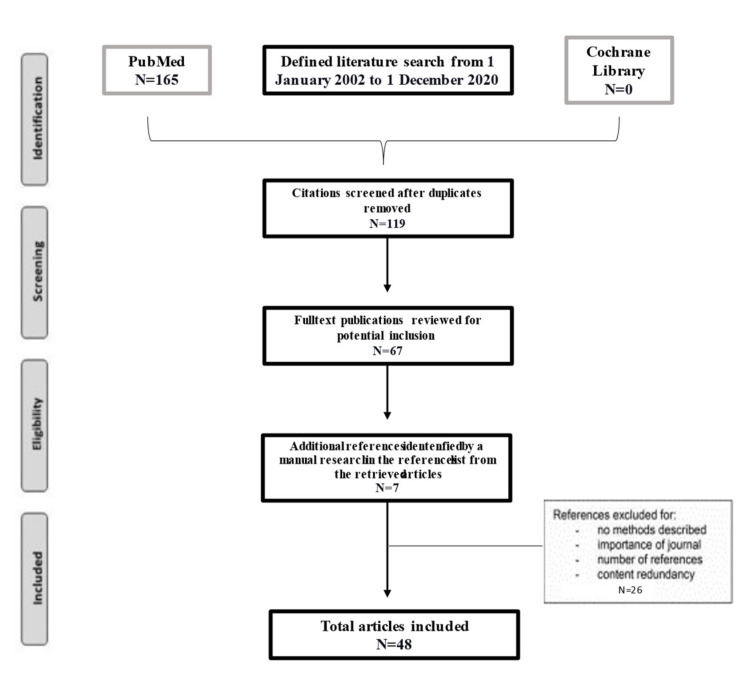
Literature narrative review flowchart.

A literature search was conducted using the PubMed database and the Cochrane library. The search terms utilized for the literature search were “bloodstream infections”, “pneumonia”, “infective endocarditis”, “Ceftobiprole”, and “COVID”. The MeSH terms were (“Ceftobiprole” [All Fields] AND “bloodstream infections” [All Fields]) OR (“Ceftobiprole” [All Fields] AND “infective endocarditis” [All Fields] OR “Ceftobiprole” [All Fields] AND “pneumonia” [All Fields]). A search period of 1 January 2002 to 1 December 2020 was selected, allowing the researchers to narratively review studies from different decades (Figure 2). Forty-eight articles were found that support the clinical efficacy of ceftobiprole in the treatment of GPB and GNB infections.
